# Renoprotective Effects of Brown-Strain *Flammulina velutipes* Singer in Chronic Kidney Disease-Induced Mice Through Modulation of Oxidative Stress and Inflammation and Regulation of Renal Transporters

**DOI:** 10.3390/ijms252212096

**Published:** 2024-11-11

**Authors:** Min-Min Lee, Yun-Xuan Chou, Sheng-Hsiung Huang, Hsu-Tang Cheng, Chung-Hsiang Liu, Guan-Jhong Huang

**Affiliations:** 1 Department of Food Nutrition and Healthy Biotechnology, College of Medical and Health Sciences, Asia University, Taichung 413, Taiwan; leemm@asia.edu.tw (M.-M.L.); sweet103203@gmail.com (Y.-X.C.); hsutangcheng@gmail.com (H.-T.C.); 2 Department of Healthcare Administration, Asia University, Taichung 413, Taiwan; harry62910@hotmail.com; 3 Department of Surgery, Asia University Hospital, Taichung 413, Taiwan; 4 Department of Neurology, China Medical University Hospital, China Medical University, Taichung 404, Taiwan; greengen@gmail.com; 5 Department of Chinese Pharmaceutical Sciences and Chinese Medicine Resources, College of Chinese Medicine, China Medical University, Taichung 404, Taiwan

**Keywords:** brown-strain *Flammulina velutipes*, cisplatin, chronic kidney disease, gallic acid, quercetin

## Abstract

Cisplatin, widely used in chemotherapy, acts through mechanisms such as oxidative stress to damage the DNA and cause the apoptosis of cancer cells. Although effective, cisplatin treatment is associated with considerable side effects including chronic kidney disease (CKD). Studies on brown-strain *Flammulina velutipes* Singer (FVB) have shown its significant antioxidant and immunomodulatory effects. High-performance liquid chromatography (HPLC) confirmed that the FVB extract contained gallic acid and quercetin. This study investigated whether FVB extract can improve and protect against cisplatin-induced CKD in mice. C57BL/6 mice were used as an animal model, and CKD was induced through intraperitoneal cisplatin injection. FVB was orally administered to the mice for 14 consecutive days. N-acetylcysteine (NAC) was administered in the positive control group. Organ pathology and serum biochemical analyses were conducted after the mice were sacrificed. Significant dose-dependent differences were discovered in body mass, kidney mass, histopathology, renal function, inflammatory factors, and antioxidant functions among the different groups. FVB extract reduced the severity of cisplatin-induced CKD in pathways related to inflammation, autophagy, apoptosis, fibrosis, oxidative stress, and organic ion transport proteins; FVB extract, thus, displays protective physiological activity in kidney cells. Additionally, orally administered high doses of the FVB extract resulted in significantly superior renal function, inflammatory factors, antioxidative activity, and fibrotic pathways. This study establishes a strategy for future clinical adjunctive therapy using edible-mushroom-derived FVB extract to protect kidney function.

## 1. Introduction

Cancer is the second leading cause of death worldwide. Although some patients recover after cancer treatment, the outcomes are not positive for many others, primarily because treatment is interrupted as a result of the toxicity of chemotherapy drugs [1,2]. Cisplatin [cis-diammine dichloro-platinum (II)] is a heavy metal platinum compound and a commonly used antitumor drug. It is administered intravenously, either as a single agent or in combination with other medications, to treat various solid organ cancers [3,4,5,6]. Cisplatin has a therapeutic effect because it forms interstrand and intrastrand DNA crosslinks in highly proliferative tumor cells, and these crosslinks lead to cell necrosis and apoptosis. Although it is effective in chemotherapy, long-term use results in numerous side effects. Nephrotoxicity is the primary dose-limiting adverse effect of cisplatin-receiving patients with cancer [7,8]. Clinical data indicate that prolonged cisplatin use leads to its accumulation in tissues of the kidneys, the primary excretion pathway; it damages renal structures and causes renal atrophy and function decline. Severe chronic kidney disease (CKD) resulting from cisplatin treatment often persists even after treatment has been successful, and complete protection of renal function and structure is unachievable [8,9]. Using repeated low-dose cisplatin (RLDC) treatment as a model for cisplatin-induced acute kidney injury (AKI)-to-CKD progression, this study shows that RLDC triggers persistent inflammatory responses in renal tubules and cultured proximal tubular cells, leading to cellular senescence and fibrosis-related maladaptive repair [10].

The nephrotoxicity of many therapeutic drugs can exacerbate deterioration in renal function, and cisplatin is a key drug inducing nephrotoxicity-related acute and chronic kidney injury. Cisplatin-induced AKI, particularly when severe and recurrent, is known to increase the risk of CKD. Clinically, patients are administered cisplatin in multiple doses weekly or monthly [11]. The proximal tubular cells are the main site of nephrotoxicity, where cisplatin reacts with glutathione and cysteine to produce reactive thiols [12]. Accumulation of cisplatin in the mitochondria of renal tubular epithelial cells causes mitochondrial structural damage, elevated reactive oxygen species (ROS) levels, and reduced activities of glutathione and superoxide dismutase (SOD). Excess ROS production worsens redox imbalance, oxidative stress, and renal damage in CKD [13]. However, renal fibrosis is the most common pathological feature in CKD patients with end-stage renal failure, marked by nephron loss, fibroblast proliferation, extracellular matrix accumulation, and tubular epithelial–mesenchymal transition (EMT). Studies have shown that transforming growth factor (TGF)-β1 is a key EMT regulator, while Smads signaling activation is crucial in promoting EMT and fibrosis [14].

The renal proximal tubule contains a transporter-mediated secretion pathway for organic anions and cations, actively facilitating the secretion of various endogenous and exogenous organic ions. Platinum derivatives are intracellularly active but their hydrophilic properties require specialized transporters to cross the cell membrane. Transporter-mediated uptake of cisplatin is critical for achieving the necessary intracellular levels, affecting both its efficacy and side effects in target and nontarget cells [15]. In nephrotoxicity models, renal tubular cation clearance significantly dropped, directly correlating with the decreased expression of organic anion transporters (OATs) and organic cation transporters (OCTs). OCTs and OATs are vital cation transporters that biological systems employ to clear harmful metabolites, ensuring efficient renal clearance of xenobiotics, toxins, and pharmaceuticals. Clinically, repeated low-dose cisplatin treatments aim to reduce side effects while maintaining anticancer efficacy [16].

Brown-strain *Flammulina velutipes* (Fr.) Singer (denoted FVB), commonly known as golden Nameko mushroom or Jinhua mushroom, is a type of brown enoki mushroom. In one study, FVB extract was reported to have excellent antioxidant activity [17]. Studies have shown that FVB is rich in quercetin and gallic acid, both of which have demonstrated kidney-protective properties [18]. The administration of quercetin significantly reduced creatinine (CRE), blood urea nitrogen (BUN), and inflammation in cisplatin-induced AKI mice, and gallic acid helped to mitigate oxidative stress, inflammation, and mitochondrial dysfunction in this acute nephrotoxicity model [19]. Therefore, FVB extract can significantly attenuate cisplatin-induced AKI and improve serum CRE and BUN levels, with similar results to the renoprotective agent amifostine [20]. To the best of our knowledge, no previous studies have investigated the effects of FVB on CKD or renal fibrosis. This research aimed to evaluate FVB’s impact on a mouse model of cisplatin-induced CKD and examine the pathways involved.

## 2. Results

### 2.1. Effect of FVB on Kidney Mass in Mice with Cisplatin-Induced Nephrotoxicity

**Figure 1:**
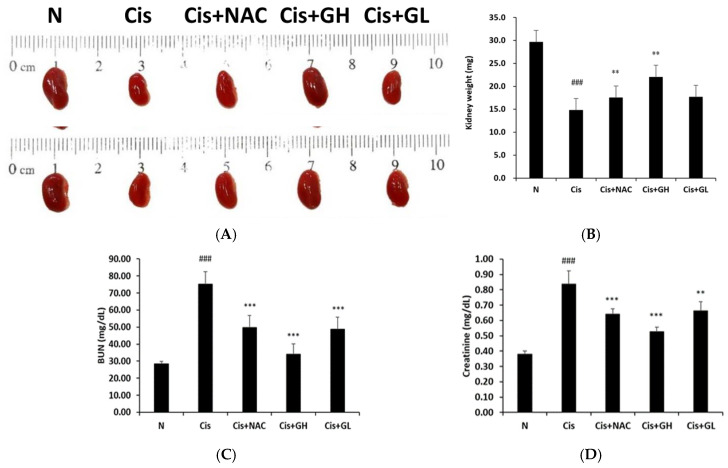
(**A**) Kidney sizes and (**B**) average kidney masses, (**C**) blood BUN nitrogen, and (**D**) CRE levels in mice with cisplatin-induced CKD. Values are presented as the means ± SD (*n* = 6). ^###^ *p* < 0.001, significant difference between the Cis and N groups; *** *p* < 0.001, and ** *p* < 0.01, significant differences between the Cis group and Cis + NAC, Cis + GL, and Cis + GH groups.

The sizes of the kidneys in the Cis group were markedly smaller than those in the N group. In the Cis + NAC, Cis + GL, and Cis + GH groups, the kidneys were less damaged than those in the Cis group, with GH having the most significant effect (Figure 1A). The average kidney mass in the Cis group was 14.8 mg lower than that in the N group (*p* < 0.001). The Cis + NAC group and Cis + GH group had kidneys with masses of 2.7 mg (*p* < 0.01) and 7.2 mg (*p* < 0.01) higher, on average, compared with the Cis group. Additionally, the kidney masses in the Cis + GH group were 4.5 mg higher than those in the Cis + NAC group (*p* < 0.05; Figure 1B). The collective results indicate that treatment with FVB led to significantly larger kidney sizes and weights in the cisplatin-induced mice. In addition, effect of FVB on Body Mass in Mice with Cisplatin-Induced Nephrotoxicity (Figure S1).

### 2.2. Effects of FVB on BUN and CRE in Mice with Cisplatin-Induced Nephrotoxicity

BUN and CRE are valuable screening tests for evaluating kidney disease. High levels of BUN and CRE can mean there is a lot of waste that has not been cleared by the kidneys. To confirm the effects of FVB on renal function, serum CRE and BUN levels were measured. The Cis group displayed a significantly elevated average BUN level relative to the N group (*p* < 0.001) but the Cis + NAC, Cis + GL, and Cis + GH groups presented significantly lower levels than the Cis group (*p* < 0.001). The Cis + GH group also had significantly lower BUN levels compared to the Cis + NAC group (*p* < 0.01; Figure 1C). The Cis group displayed significantly elevated CRE levels compared to the N group (*p* < 0.001), whereas the Cis + NAC group had a notably lower average CRE level than the Cis group (*p* < 0.001). Both the Cis + GH and Cis + GL groups showed significant reductions in CRE levels, with the Cis + GH group exhibiting the greatest reduction (*p* < 0.001). The average CRE level in the Cis + GH group was significantly lower than that in the Cis + NAC group (*p* < 0.001; Figure 1D). Collectively, the data show that FVB reduced both BUN and CRE in the mice with CKD.

### 2.3. Effect of FVB on Kidney Index in Mice with Cisplatin-Induced Nephrotoxicity

The average kidney index in the Cis group was 0.32 mg/g lower than that in the N group (*p* < 0.001), and both body mass (*p* < 0.001) and kidney mass (*p* < 0.001) in the Cis group were significantly lower. The average kidney index in the Cis + NAC and Cis + GH groups significantly recovered to levels close to that of the N group; these groups had indexes of 0.21 mg/g (*p* < 0.001) and 0.33 mg/g (*p* < 0.001) higher than that of the Cis group, respectively. The Cis + GL group had an average index of 0.19 mg/g higher than that of the Cis group (*p* < 0.01). The values in the Cis + GH group were not significantly different from those in the Cis + NAC group (Table 1); however, the mice treated with FVB showed significantly higher resistance to cisplatin-induced nephrotoxicity, such as a reduction in the kidney index.

### 2.4. Effect of FVB on Renal Hematoxylin and Eosin (H&E)-Stained Sections of Mice with Cisplatin-Induced Nephrotoxicity

**Figure 2:**
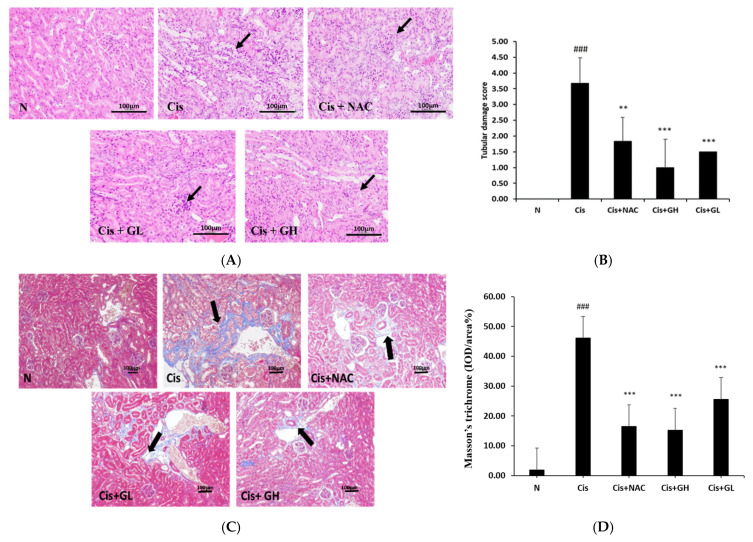
(**A**) Renal H&E-stained sections (400×), (**B**) tubular damage scores, (**C**) renal Masson’s trichrome-stained sections showing fibrosis (400×), and (**D**) tubular damage scores in mice with cisplatin-induced CKD. Values are presented as the means ± SD (*n* = 6). ^###^ *p* < 0.001, significant difference between the Cis and N groups; ** *p* < 0.01 and *** *p* < 0.001, significant differences between the Cis group and Cis + NAC, Cis + GL, and Cis + GH groups. The arrows indicate the nephron glomerulus. The bars represent 100 μm.

Cisplatin-induced nephrotoxicity was evidenced by histopathological changes such as tubular necrosis, vacuolation, desquamation of epithelial cells in renal tubules, and the presence of tubular casts. The N group showed intact and well-organized glomeruli and renal tubules. In the Cis group, the glomeruli were atrophied, the renal tubules were damaged, and the overall structures were loose and infiltrated by inflammatory substances. In the Cis + NAC group, the samples exhibited some repair, with the glomeruli and renal tubules having normal structures. Both doses of FVB extract, particularly the higher dose, resulted in better structures than those found in the Cis group. The Cis + GH group samples had well-structured glomeruli and renal tubules and overall tight structural arrangements (Figure 2A). The tubular damage scores indicate that the structural damage to tissues was most severe in the Cis group (*p* < 0.001). The amount of damage was significantly less in the Cis + NAC group (*p* < 0.01) and even more significantly reduced in the Cis + GH and Cis + GL groups (*p* < 0.001). The difference between the damage scores of the Cis + GH group and the Cis + NAC group was nonsignificant (Figure 2B). Thus, treatment with FVB improves kidney activity and structure in mice with cisplatin-induced CKD.

### 2.5. Effect of FVB on Renal Masson’s Trichrome-Stained Sections of Mice with Cisplatin-Induced Nephrotoxicity

The progression of chronic kidney disease to end-stage renal disease is primarily caused by renal fibrosis. This study utilized Masson’s trichrome staining for fibrosis analysis. Compared with tissues from the N group, those of the Cis group were fragile, exhibited extensive blue fibrosis, and had hollow and loose structures. Mild fibrosis was discovered in the Cis + GL group but the level of fibrosis was significantly less in the Cis + GH and Cis + NAC groups, with the samples from these groups retaining their overall structural integrity (Figure 2C). Statistical analysis revealed that the fibrosis in the Cis group was significantly more extensive than that in the N group (*p* < 0.001). The Cis + NAC, Cis + GL, and Cis + GH groups had significantly less fibrosis than the Cis group (*p* < 0.001). The difference between the fibrosis in the Cis + GH and Cis + NAC groups was nonsignificant (Figure 2D). Thus, treatment with FVB improves kidney fibrosis in mice with cisplatin-induced CKD.

### 2.6. Effects of FVB on Glutathione (GSH), Nitrite, and Malondialdehyde (MDA) in Kidneys with Cisplatin-Induced Nephrotoxicity

**Figure 3:**
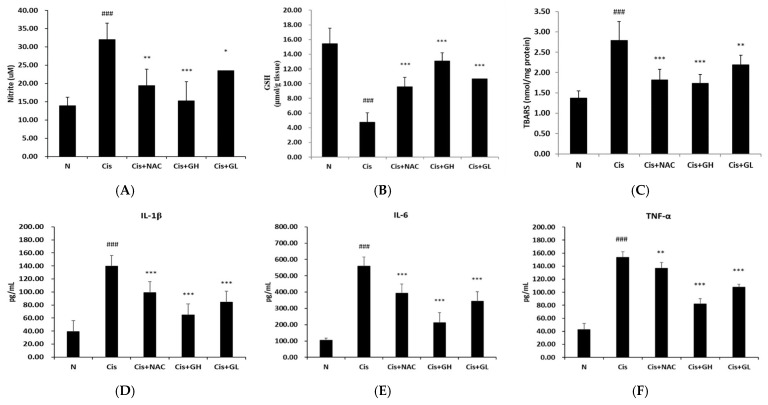
Levels of (**A**) glutathione, (**B**) nitrite, and (**C**) thiobarbituric acid reactive substance (TBARS) as well as (**D**) IL-1β, (**E**) IL-6, and (**F**) TNF-α inflammatory cytokines in mice with cisplatin-induced CKD. Values are presented as the means ± SD (*n* = 6). ^###^ *p* < 0.001, significant difference between the Cis and N groups; * *p* < 0.05, ** *p* < 0.01, and *** *p* < 0.001, significant differences between the Cis group and Cis + NAC, Cis + GL, and Cis + GH groups.

Oxidative stress is involved as a mechanism of cisplatin-induced kidney injury. Glutathione is a major component of the cellular antioxidant defense system and performs important physiological functions. The N group had normally high levels of antioxidant function, as measured using GSH levels. The Cis group had a significantly lower average GSH level than the N group (*p* < 0.001) and the Cis + NAC group (*p* < 0.001). Additionally, the Cis + GL and Cis + GH groups had higher average GSH levels than the Cis group (*p* < 0.001), with the average level of the Cis + GH group also being higher than the Cis + NAC group (*p* < 0.01; Figure 3A).

Cisplatin-induced inflammation triggers the upregulation of inducible nitric oxide synthase (iNOS), which directly promotes nitric oxide (NO) production. The inflammatory response was indicated by the nitrite level, which was low in the N group but significantly higher in the Cis group (*p* < 0.001). The Cis + NAC group had a significantly lower average nitrite level than the Cis group (*p* < 0.01) and the same was true for the Cis + GH group (*p* < 0.001) and Cis + GL group (*p* < 0.05). The difference in average nitrite levels between the Cis + GH and Cis + NAC groups was found to be nonsignificant (Figure 3B).

ROS target cell membrane lipids, causing peroxidation and protein denaturation. Cisplatin treatment stimulates ROS production, contributing to CKD development. The average level of MDA, a product of lipid peroxidation, was significantly higher in the Cis group than the N group (*p* < 0.001). The Cis + NAC, Cis + GH, and Cis + GL groups had lower average MDA levels than the Cis group (*p* < 0.001, 0.001, and 0.01, respectively). The difference between the Cis + NAC and Cis + GH groups was nonsignificant (Figure 3C). Thus, FVB treatment can reduce oxidative stress in cisplatin-induced nephrotoxic mice.

### 2.7. Effects of FVB on Cytokine Levels in Mice with Cisplatin-Induced Nephrotoxicity

TNF-α, IL-1β, and IL-6 are proinflammatory cytokines known to play important roles in cisplatin-induced renal injury. Therefore, we evaluated serum TNF-α, IL-1β, and IL-6 concentrations by ELISA after cisplatin injection. The inflammatory cytokine IL-1β had significantly higher levels on average in the Cis group than in the N group (*p* < 0.001). However, the IL-1β levels in the Cis + NAC, Cis + GL, and Cis + GH groups were considerably lower compared to the Cis group (all *p* < 0.001), with the Cis + GH group having a significantly lower average level than the Cis + NAC group (*p* < 0.001; Figure 3D). The same results and significance levels were obtained for the IL-6 levels. Both doses of FVB extract led to nearly normal IL-6 levels. The Cis + GH group had a lower average IL-6 level than the Cis + NAC group (*p* < 0.001; Figure 3E). In the results for inflammatory TNF-α, the levels were, again, significantly higher in the Cis group than the N group (*p* < 0.001). The Cis + NAC group exhibited a significantly lower average TNF-α level compared to the Cis group (*p* < 0.01), with the Cis + GL and Cis + GH groups showing even more pronounced reductions (*p* < 0.001). Additionally, the Cis + GH group had a significantly lower average TNF-α level than the Cis + NAC group (*p* < 0.001; Figure 3F). Collectively, the data demonstrate that FVB resulted in significant decreases in pro-inflammatory cytokine levels.

### 2.8. Effects of FVB on Inducible Nitric Oxide Synthase (iNOS) and Cyclooxygenase-2 (COX-2) in Mice with Cisplatin-Induced Nephrotoxicity

**Figure 4:**
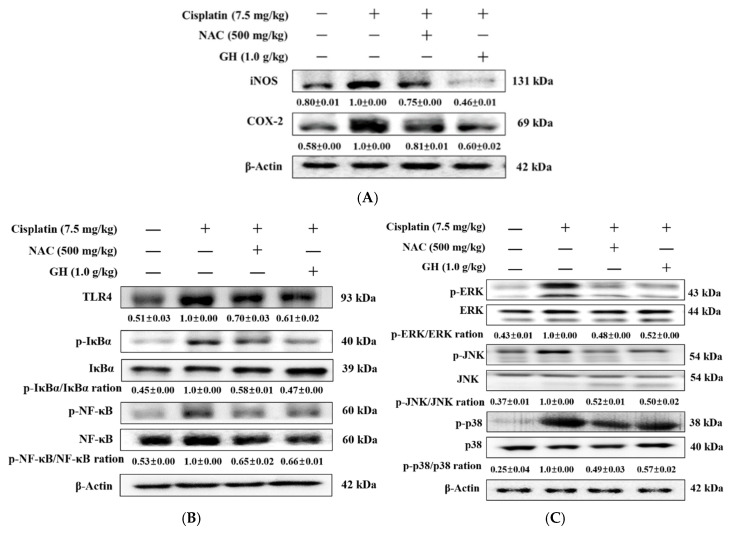
(**A**) Inducible nitric oxide synthase (iNOS) and cyclooxygenase-2 (COX-2); (**B**) TLR4, IκBα, and NF-κB; and (**C**) MAPKs levels in mice with cisplatin-induced CKD. The statistical analysis of these levels involved triplicate measurements. Values are expressed as the means ± SD. The bands were quantified using the densitometric program ImageJ (version 1.8.0); the protein levels were then divided by β-actin and normalized against the control protein.

To investigate if FVB protects renal cells from cisplatin-induced inflammation, the proteins iNOS and COX-2, both linked to inflammation, were analyzed. The Cis group displayed significantly elevated average iNOS and COX-2 expression levels compared to the N group (*p* < 0.001) but these levels were significantly reduced in both the Cis + NAC and Cis + GH groups (*p* < 0.001). The Cis + GH group showed even lower average iNOS and COX-2 expression levels than the Cis + NAC group (*p* < 0.01 and 0.001, respectively; Figure 4A). The results reveal that treatment with FVB inhibited the protein expression of iNOS and COX-2 in kidney tissues after the cisplatin challenge.

### 2.9. Effects of FVB on TLR4, IκBα, and NF-κB Levels in Mice with Cisplatin-Induced Nephrotoxicity

To investigate if FVB protects renal cells from cisplatin-induced inflammation, the proteins iNOS and COX-2, both linked to inflammation, were analyzed. TLR4 levels were low in the N group but increased significantly in the Cis group (*p* < 0.001). The Cis + NAC and Cis + GH groups had significantly lower average TLR4 levels than the Cis group (both *p* < 0.001), with the levels in the Cis + GH group being lower than those in the Cis + NAC group (*p* < 0.05). The Cis group had a significantly higher average level of IκBα than the N group (*p* < 0.001), whereas the Cis + NAC and Cis + GH groups had significantly lower average IκBα levels than the Cis group (both *p* < 0.001). The data also reveal a significant difference between these levels in the Cis + GH and Cis + NAC groups, with significantly lower levels in the Cis + GH group (*p* < 0.01). The average NF-κB level was significantly higher in the Cis group than in the N group (*p* < 0.001). The Cis + NAC and Cis + GH groups both had significantly lower average NF-κB levels than the Cis group (both *p* < 0.001). The difference between the Cis + GH and Cis + NAC groups for this level was nonsignificant (Figure 4B). Thus, FVB regulates the TLR4/NF-κB signaling pathway after the cisplatin challenge.

### 2.10. Effects of FVB on Extracellular Signal-Regulated Kinase (ERK), c-Jun NH2-Terminal Kinase (JNK), and p38 in Mice with Cisplatin-Induced Nephrotoxicity

We used Western blotting to investigate the protein expression levels in the MAPK signaling pathway to better understand the molecular mechanisms through which FVB protects against cisplatin-induced kidney damage. ERK, JNK, and p38 levels, which are linked to inflammation, were significantly elevated in the Cis group relative to the N group (*p* < 0.001). Conversely, the Cis + NAC and Cis + GH groups had significantly reduced levels of these proteins compared to the Cis group (*p* < 0.001). Significant differences in ERK and p38 levels were observed between the Cis + GH and Cis + NAC groups, with the Cis + NAC group showing lower levels (*p* < 0.05). No significant differences were detected in the JNK levels between these two groups (Figure 4C). These results indicate that FVB regulates the TLR4/MAPK/NF-κB pathway in cisplatin-treated CKD.

### 2.11. Effects of FVB on Beclin-1, Light Chain 3 (LC3)-I/II, and p62 in Mice with Cisplatin-Induced Nephrotoxicity

**Figure 5:**
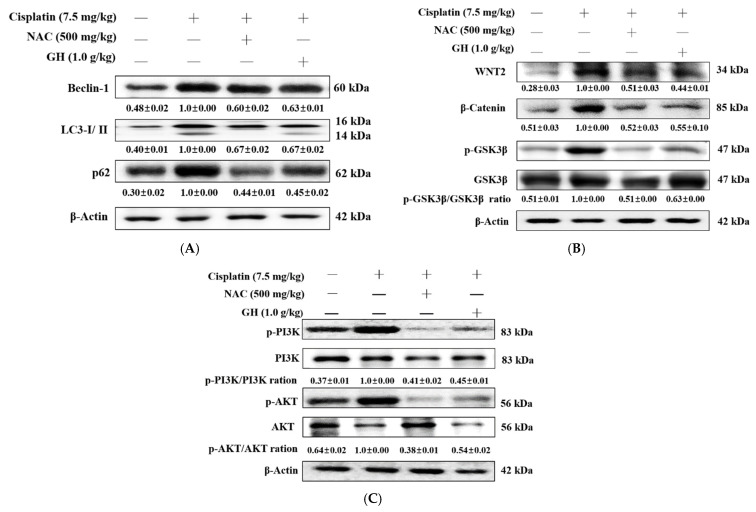
(**A**) Beclin-1, LC3-I/II, and p62, (**B**) WNT2, β-catenin, and GSK3β, and (**C**) PI3K and AKT levels in the N, Cis, Cis + NAC, and Cis + GH groups of mice with cisplatin-induced chronic kidney disease. The statistical analysis of these levels involved triplicate measurements. Values are expressed as the means ± SD. The bands were quantified using the densitometric program ImageJ; the protein levels were then divided by β-actin and normalized against the control protein.

This research analyzed biochemical markers from autophagy pathways to explore their impact on kidney injury in mice. Beclin-1, LC3-I/II, and p62, which are related to autophagy, exhibited significantly higher average levels in the Cis group compared to the N group (all *p* < 0.001). In contrast, the average levels of these proteins were significantly reduced in the Cis + NAC and Cis + GH groups compared to the Cis group (all *p* < 0.001). No significant differences were observed between the Cis + NAC and Cis + GH groups (Figure 5A). These results demonstrate that FVB improved the expression of autophagy-related proteins in cisplatin-treated CKD.

### 2.12. Effects of FVB on Wnt/β-catenin/Glycogen Synthase Kinase-3β (GSK3β) in Mice with Cisplatin-Induced Nephrotoxicity

Protein expression in the Wnt/β-catenin/GSK3β pathway was analyzed via Western blotting to explore how FVB reduces cisplatin-induced nephrotoxicity. Appropriate autophagy supports cell survival but excessive autophagy can cause adverse cellular functioning. The Wnt/β-catenin/GSK3β pathway regulates cell growth, differentiation, and survival. Dysregulation of this pathway is implicated in cancers and fibrotic diseases. The average WNT2, β-catenin, and GSK3β protein expression levels were low in the N group; however, these levels were significantly elevated in the Cis group compared to the N group (*p* < 0.001). The Cis + NAC and Cis + GH groups had significantly lower average WNT2, β-catenin, and GSK3β levels than the Cis group (*p* < 0.001). The difference in the average WNT2 levels between the Cis + GH and Cis + NAC groups was nonsignificant but the average β-catenin and GSK3β levels were significantly lower in the Cis + NAC group than in the Cis + GH group (*p* < 0.001; Figure 5B). These results demonstrate that FVB regulates the Wnt/β-catenin/GSK3β axis in cisplatin-treated CKD.

### 2.13. Effects of FVB on PI3K and AKT in Mice with Cisplatin-Induced Nephrotoxicity

Protein expression in the PI3K/AKT pathway was assessed via Western blotting to investigate how FVB protects against cisplatin-induced renal injury. PI3K, a marker for the apoptotic pathway, was significantly more expressed in the Cis group than in the N group (*p* < 0.001). Both the Cis + NAC and Cis + GH groups showed significantly lower average PI3K levels compared to the Cis group (*p* < 0.001), though no significant difference was found between these two groups. AKT expression was also markedly elevated in the Cis group relative to the N group (*p* < 0.001) but significantly reduced in the Cis + NAC and Cis + GH groups compared to the Cis group (*p* < 0.001). Notably, AKT levels were significantly lower in the Cis + NAC group than in the Cis + GH group (*p* < 0.001; Figure 5C). These results demonstrate that FVB regulates the PI3K/AKT axis in cisplatin-treated CKD.

### 2.14. Effects of FVB on p53, Bax, Bcl-2, and Caspase-3 in Mice with Cisplatin-Induced Nephrotoxicity

**Figure 6:**
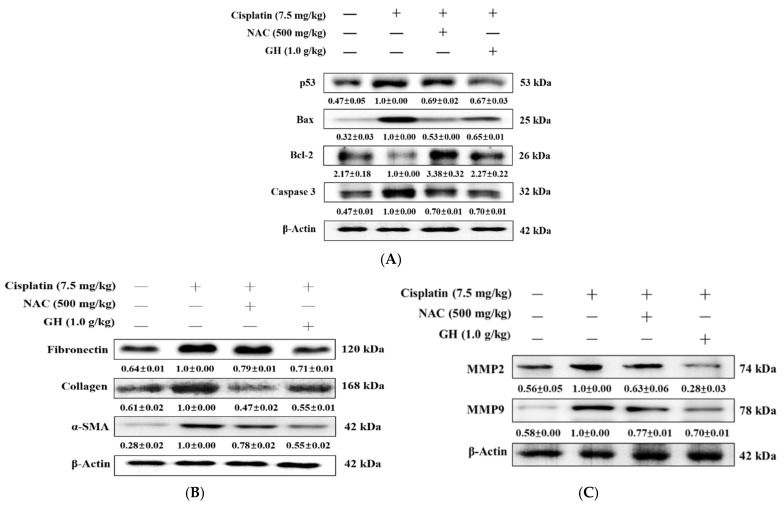
(**A**) p53, Bax, Bcl-2, and caspase-3, (**B**) TGF-β and SMAD3, and (**C**) matrix metalloproteinase 2 (MMP2) and MMP9 levels in mice with cisplatin-induced CKD. The statistical analysis of these levels involved triplicate measurements. Values are expressed as the means ± SD. The bands were quantified using the densitometric program ImageJ; the protein levels were then divided by β-actin and normalized against the control protein.

The biochemical markers of apoptosis signaling pathways were analyzed in this study to explore their involvement in mouse kidney damage. Regarding the apoptotic pathway, the average p53 level in the Cis group was significantly higher than that in the N group (*p* < 0.01). The Cis + GH and Cis + NAC groups had significantly lower average p53 levels than the Cis group (*p* < 0.01 and 0.001, respectively). The difference in the p53 levels between the Cis + GH and Cis + NAC groups was nonsignificant. Reciprocal regulation was discovered between the key apoptotic regulators Bax (proapoptotic) and Bcl-2 (antiapoptotic); the Cis group had a significantly higher average protein Bax level and significantly lower average protein Bcl-2 level than those of the N group (both *p* < 0.001). Both the Cis + NAC and Cis + GH groups showed significantly lower average Bax levels (*p* < 0.001) and higher average Bcl-2 levels (*p* < 0.001) compared with the Cis group. Furthermore, the average Bax level was notably lower in the Cis + NAC group compared to the Cis + GH group (*p* < 0.05), whereas the average Bcl-2 level was significantly elevated in the Cis + NAC group relative to the Cis + GH group (*p* < 0.001). The Cis group exhibited a significantly higher average caspase-3 level than the N group (*p* < 0.001) but the Cis + NAC and Cis + GH groups had significantly lower levels compared to the Cis group (*p* < 0.01 and 0.001, respectively). The difference in caspase-3 levels between the Cis + GH and Cis + NAC groups was nonsignificant (Figure 6A). These results demonstrate that FVB improved the expression of apoptosis-related proteins in cisplatin-treated CKD.

### 2.15. Effects of FVB on Transforming Growth Factor-β (TGF-β) and SMAD3 in Mice with Cisplatin-Induced Nephrotoxicity

Western blot analysis was applied to assess protein expression in the TGF-β/Smad3 signaling pathway, exploring how FVB protects against cisplatin-induced kidney injury. The average level of TGF-β, a key protein in apoptosis and fibrosis, was significantly higher in the Cis group than in the N group (*p* < 0.001) but significantly lower in the Cis + NAC and Cis + GH groups than in the Cis group (*p* < 0.001). A significant difference in these levels was discovered between the Cis + GH and Cis + NAC groups, with that of the Cis + GH group being lower (*p* < 0.001). The average level of the fibrosis-related protein SMAD3 was significantly higher in the Cis group than in the N group (*p* < 0.001) and significantly lower in the Cis + NAC and Cis + GH groups than in the Cis group (*p* < 0.001). The difference in these levels between the Cis + GH and Cis + NAC groups was nonsignificant (Figure 6B). These results demonstrate that FVB regulates the TGF-β/Smad3 axis in cisplatin-treated CKD.

### 2.16. Effects of FVB on Matrix Metalloproteinase 2 (MMP2) and MMP9 in Mice with Cisplatin-Induced Nephrotoxicity

To explore how FVB influences cisplatin-induced nephrotoxicity, we performed Western blot analysis to evaluate MMP2 and MMP9 protein expression levels. The average levels of the fibrosis-related proteins MMP2 and MMP9 were significantly higher in the Cis group compared with the N group (*p* < 0.001). Compared with the Cis group, the Cis + NAC and Cis + GH groups had significantly lower average MMP2 and MMP9 expression levels (Cis + NAC: *p* < 0.01 and 0.001, respectively; Cis + GH: both *p* < 0.001). Compared with the Cis + NAC group, the Cis + GH group had significantly lower average MMP2 and MMP9 levels (*p* < 0.01 and 0.001, respectively; Figure 6C). These results demonstrate that FVB reduced the expression of the MMP2 and MMP9 proteins in cisplatin-treated CKD.

### 2.17. Effects of FVB on Fibronectin, Collagen, and α-Smooth Muscle Actin (SMA) in Mice with Cisplatin-Induced Nephrotoxicity

**Figure 7:**
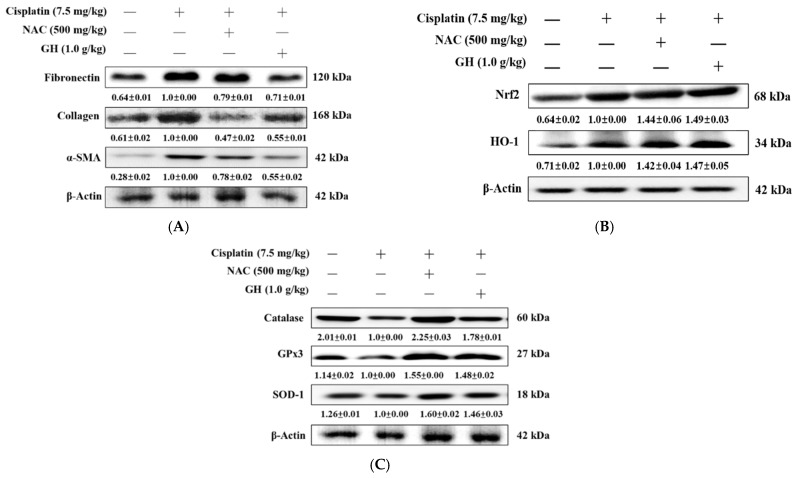
(**A**) Fibronectin, collagen, and α-smooth muscle actin (SMA), (**B**) nuclear factor erythroid–related factor 2 (Nrf2) and heme oxygenase-1 (HO-1), and (**C**) catalase, glutathione peroxidase 3 (GPx3), and superoxide dismutase type 1 (SOD-1) levels in mice with cisplatin-induced CKD. The statistical analysis of these levels involved triplicate measurements. Values are expressed as the means ± SD. The bands were quantified using the densitometric program ImageJ; the protein levels were then divided by β-actin and normalized against the control protein.

Fibrosis-related proteins were detected to evaluate whether FVB could protect renal cells from cisplatin-induced chronic renal fibrosis. The fibronectin, collagen, and α-SMA levels in the Cis group were significantly higher than those in the N group (all *p* < 0.001), whereas the Cis + NAC and Cis + GH groups showed significantly lower levels than those in the Cis group (*p* < 0.001). The Cis + GH group had significantly lower fibronectin and α-SMA levels than the Cis + NAC group (*p* < 0.05 and *p* < 0.001) but collagen was significantly lower in the Cis + NAC group (*p* < 0.05; Figure 7A). These results demonstrate that FVB reduced the expression of fibrosis-related proteins in cisplatin-treated CKD.

### 2.18. Effects of FVB on Nuclear Factor Erythroid–Related Factor 2 (Nrf2) and Heme Oxygenase-1 (HO-1) in Mice with Cisplatin-Induced Nephrotoxicity

In order to investigate how FVB reduces kidney injury caused by cisplatin, the expression levels of the HO-1/Nrf2 axis were detected through Western blotting. Nrf2, a key protein in the cellular stress response, and HO-1, its downstream effector, were significantly upregulated in the Cis group compared to the N group (*p* < 0.001 and *p* < 0.01, respectively). The Cis + NAC and Cis + GH groups had significantly higher Nrf2 levels than the Cis group (*p* < 0.001) without a significant difference between them. The Cis + NAC and Cis + GH groups also had significantly higher HO-1 levels compared to the Cis group (*p* < 0.001). The difference between the levels of the Cis + GH and Cis + NAC groups was nonsignificant (Figure 7B). These results demonstrate that FVB regulates the HO-1/Nrf2 axis in cisplatin-treated CKD.

### 2.19. Effects of FVB on Catalase, Glutathione Peroxidase 3 (GPx3), and Superoxide Dismutase Type 1 (SOD-1) in Mice with Cisplatin-Induced Nephrotoxicity

To examine whether FVB alters antioxidant enzymes to help protect renal cells from the effects of cisplatin-induced CKD. The average level of catalase, which is involved in antioxidant function, was significantly lower in the Cis group than in the N group (*p* < 0.001). The Cis + NAC and Cis + GH groups had significantly higher average catalase levels than the Cis group (*p* < 0.001), and the Cis + NAC group had a significantly higher average level than the Cis + GH group (*p* < 0.001). The Cis group displayed a significantly elevated average GPx3 level compared to the N group (*p* < 0.001). Both the Cis + NAC and Cis + GH groups had significantly higher GPx3 levels than the Cis group (*p* < 0.001), with no notable difference between the Cis + NAC and Cis + GH groups. The SOD-1 protein expression was significantly reduced in the Cis group compared to the N group (*p* < 0.01) but the levels in the Cis + NAC and Cis + GH groups were significantly higher than that in the Cis group (*p* < 0.01 and 0.001, respectively). Furthermore, the Cis + NAC group had a significantly higher level than the Cis + GH group (*p* < 0.05; Figure 7C). These results demonstrate that FVB reduced the expression of antioxidative-related proteins in cisplatin-treated CKD.

### 2.20. Effects of FVB on Organic Cation Transporters (OCTs) and Organic Anion Transporters (OATs) in Mice with Cisplatin-Induced Nephrotoxicity

**Figure 8:**
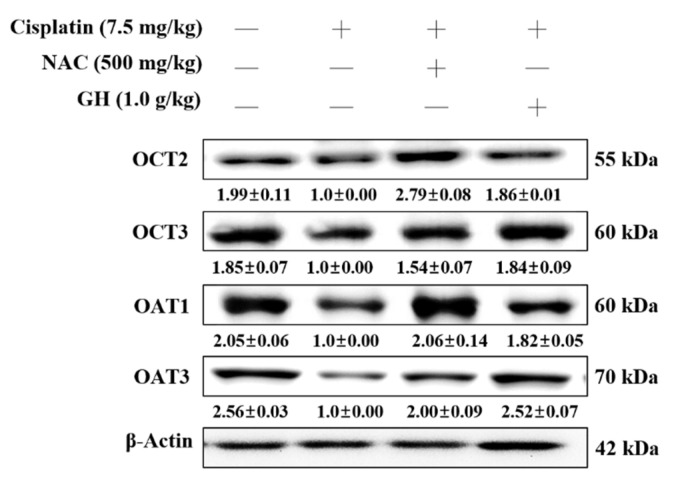
Levels of organic cation transport proteins (OCT2 and OCT3) and organic anion transport proteins (OAT1 and OAT3) in mice with cisplatin-induced CKD. The statistical analysis of these levels involved triplicate measurements. Values are expressed as the means ± SD. The bands were quantified using the densitometric program ImageJ; the protein levels were then divided by β-actin and normalized against the control protein.

We investigated the potential involvement of FVB with transporters in the protection of renal cells from cisplatin-induced CKD. The average levels of OCT2 and OCT3 were significantly lower in the Cis group than in the N group (*p* < 0.001). The Cis + NAC and Cis + GH groups had significantly higher average OCT2 (both *p* < 0.001) and OCT3 (*p* < 0.01 and 0.001, respectively) levels than the Cis group. A statistical difference in OCT2 levels was observed between the Cis + GH and Cis + NAC groups, with the Cis + NAC group having a significantly higher level of OCT2 (*p* < 0.01) but significantly lower level of OCT3 (*p* < 0.001) than the Cis + GH group. The Cis group exhibited significantly lower average levels of OAT1 and OAT3 compared to the N group (*p* < 0.001). The Cis + GH and Cis + NAC groups both had significantly elevated levels of these proteins compared to the Cis group (*p* < 0.001 for OAT1; *p* < 0.01 for OAT3). OAT1 was expressed at significantly higher levels in the Cis + NAC group than in the Cis + GH group (*p* < 0.05) but OAT3 expression was significantly higher in the Cis + GH group (*p* < 0.05; Figure 8). These results demonstrate that FVB increased the expression of OCT2, OCT3, OAT1, and OAT3 proteins in cisplatin-treated CKD.

### 2.21. Qualitative and Quantitative Analyses of FVB Extract by HPLC

**Figure 9:**
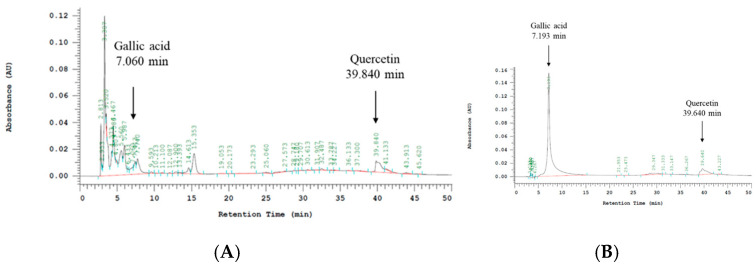
HPLC results for the FVB extract: (**A**) detection of gallic acid at a retention time of 7.060 min and quercetin at 39.840 min in the analysis of the FVB extract measured at 20,000 μg/g; (**B**) detection of gallic acid at a retention time of 7.193 min and quercetin at 39.640 min in the analysis of 100 μg/g standard samples.

Gallic acid and quercetin were identified as bioactive components in the FVB extract [20]. In the present study, the HPLC analysis detected gallic acid at a retention time of 7.060 min, with a relative concentration of 8.83 μg/g corresponding to 29.43 μg per 100 mg of FVB extract. Quercetin was detected at a retention time of 39.840 min, with a relative concentration of 66.93 μg/g, corresponding to 334.65 μg per 100 mg of FVB extract (Figure 9).

## 3. Discussion

This study tested cultivated FVB by evaluating its potential to effectively mitigate the side effects of cisplatin in CKD. The findings indicate that the FVB extract provides significant antioxidant effects [17] and can help relieve cisplatin-induced AKI [20]. The present study further investigated the extract’s efficacy in a cisplatin-induced CKD animal model by conducting various biochemical assays and using molecular biological mechanisms as indicators.

Long-term use of cisplatin results in notable changes in the kidneys’ function and structure. This study discovered that compared with the N group, the Cis group exhibited kidney atrophy (in terms of appearance and size) and had a lower average body mass and a lower kidney-to-body-mass ratio; additionally, in H&E-stained histological sections of the mice in this group, glomerular and tubular structures were damaged and had loose tissue arrangements. Masson’s trichrome-stained micrographs revealed severe tissue fibrosis with extensive blue-stained fibrous areas and structural voids. Functionally, the Cis group had significantly higher serum BUN and CRE levels than the N group, indicating that renal excretion had been impaired because of the cisplatin. The intervention with the FVB extract significantly ameliorated the cisplatin-induced damage, with the high Cis dose (1.0 g/kg) being the most effective. The extract was also discovered to outperform the clinically used adjuvant NAC in terms of body mass, kidney improvement, and BUN and CRE levels; it brought the kidney structure and function closer to normal levels and most effectively mitigated cisplatin-induced renal damage.

Inflammatory responses can trigger a considerable increase in cell-damaging substances [21]. Cisplatin induces inflammatory pathways, elevating the risk of CKD. Researchers have shown that cisplatin injection significantly upregulates the TLR4/IκBα/NF-κB pathway [22,23] and the MAPK pathway protein expression [16], leading to increased levels of proinflammatory factors such as COX-2, iNOS, IL-1β, IL-6, TNF-α, and nitrites in kidney tissue [24]. TLRs are involved in inflammation and innate immunity. Increased TLR4 protein expression activates the TLR4 adapter protein MyD88, which stimulates TRAF6 and IRAK1, leading to an inflammatory response and increased NF-κB activity. Normally, NF-κB is bound to IκB in the cytoplasm, but under stress conditions, IκB is phosphorylated, releasing NF-κB to translocate to the nucleus. Oxidative stress further enhances NF-κB activation, promoting cytokine release [25]. FVB extract helps reduce inflammation and subsequent pathway progression. Compared with the NAC adjuvant, FVB extracts more effectively ameliorate inflammation-related expression of TLR4, IκBα, iNOS, COX-2, IL-1β, IL-6, and TNF-α.

Moderate autophagy is a normal clearance mechanism and promotes cell survival but excessive autophagy can lead to abnormal apoptosis in kidney cells [26,27]. Cisplatin acts as an activator of inflammation under stress conditions, producing reactive oxygen species (ROS) and cellular damage and thereby increasing the abnormal expression of Beclin-1, LC3 I/II, and p62 [28,29]. Additionally, regulation through the Wnt/β-catenin/GSK3β pathway can inhibit abnormal autophagy activation, having a protective effect on cells [30]. The Wnt/β-catenin/GSK3β signaling pathway is critical for controlling cell proliferation and migration [31]. Research shows that Wnt signaling suppression activates autophagy in colorectal cancer cells, with β-catenin binding to LC-3, thereby obstructing autophagosome development [32]. Moreover, TGF-β signals through numerous intracellular molecules, with evidence indicating the importance of the Wnt/β-catenin/GSK3β pathway in TGF-β-induced fibrosis. Blocking the Wnt/β-catenin/GSK3β pathway could help decrease fibrosis after AKI [33]. The study indicates that FVB extract has protective properties and may mitigate the harmfulness of autophagic responses.

Apoptosis is closely related to cell survival. Apoptotic factors, such as p53, Bax/Bcl-2, and caspase-3 increase when the cisplatin concentration is increased [9,34], stimulating the TGF-β/PI3K/AKT pathway to activate apoptosis [35,36]. The proapoptotic protein Bax and antiapoptotic protein Bcl-2 inhibit each other. Cisplatin increases Bax protein expression while inhibiting Bcl-2 protein expression, and this action accelerates cell death. The Bcl-2 family of proteins is essential in the regulation of the apoptosis process. These proteins, activated through the intrinsic apoptotic pathway, respond to DNA damage, oxidative stress, radiation, and nutrient deprivation. Bcl-2 acts as an antiapoptotic factor and is inversely related to Bax. In unilateral ureteral obstruction (UUO), Bcl-2 levels are diminished and the same is seen in patients with end-stage renal disease (ESRD) who undergo dialysis. The reduction in Bcl-2 promotes Bax, causing cytochrome C release and mitochondrial damage, leading to cell death and, ultimately, contributing to kidney fibrosis and CKD [34]. The present study found that the FVB extract increased Bcl-2 protein expression, blocking the subsequent pathways for cisplatin-induced apoptosis and increasing renal cell survival, providing renal cells with the opportunity for repair. Compared with NAC, the FVB extract also exhibited a more satisfactory performance in terms of TGF-β expression.

Renal fibrosis results from prolonged abnormal cell damage and excessive amounts of inflammatory substances [27,35]. Activation of the fibrosis pathway is the main cause of structural damage and atrophy in the kidneys. Through the TGF-β/SMAD3 pathway, cisplatin indirectly increases fibronectin, collagen, and α-SMA secretion and accumulation at damaged renal sites [36]. TGF-β is widely recognized as the key driver of renal fibrosis, and Smad signaling plays a central role in its fibrotic signaling. Smad3 activation and Smad7 downregulation via ubiquitin E3 ligase degradation characterize fibrogenesis. TGF-β contributes to kidney fibrosis by (1) promoting ECM production (collagen I and fibronectin) through Smad3, (2) blocking ECM degradation by inhibiting MMPs, and (3) aggravating renal damage through direct effects on renal cells [37,38]. MMP2 and MMP9 are activated and participate in the fibrosis response [39,40]. The intervention with the FVB extract significantly reduced fibrosis expression and improved fibrosis in damaged areas. Compared with the mice receiving NAC, those receiving a high dose of the FVB extract exhibited more significant inhibition of the fibrosis pathway in terms of TGF-β, MMP2, MMP9, fibronectin, and α-SMA levels.

Buildup of cisplatin in renal epithelial cells leads to cellular injury and a sharp increase in ROS [41]. This study found that the Cis group had higher levels of lipid peroxides, such as MDA, and significantly lower levels of antioxidant enzymes, including SOD-1, catalase, GPx3, and GSH. This resulted in increased oxidative stress and would lead to CKD. Obesity and hyperlipidemia are key risk factors for CKD, demonstrating that lipid accumulation in kidney tissue is harmful to renal function. Free fatty acids (FFAs) are particularly detrimental. Increased FFA uptake from overactive fatty acid transport and reduced β-oxidation leads to lipid buildup in nonadipose tissue [42]. The damage to renal tubular cells and interstitial tissue caused by excessive FFAs is due to increased ROS production, lipid peroxidation, and mitochondrial damage, which in turn cause inflammation and tissue damage. The FVB extract diminished the excessive production of MDA and elevated the levels of antioxidant enzymes, including SOD-1, catalase, GPx3, and GSH. It also activated the Nrf2/HO-1 pathway [43]. The FVB extract promoted a balance between oxidation and antioxidation, assisting in the removal of excess ROS, with effects similar to those of NAC. Disruption of the Nrf2 system and oxidative stress play vital roles in CKD development and pathogenesis. The activation of Nrf2 in CKD influences inflammation and renal fibrosis as well as mitochondrial and metabolic functions [44]. Oxidative stress also releases Nrf2 from the Nrf2–Keap1 complex, activating the expression of Nrf2 and its downstream genes, which is essential for inhibiting inflammation via antioxidant pathways. Nrf2, being a transcription factor, promotes the expression of genes encoding antioxidant-related proteins, such as HO-1, GPx, and GSH-S-transferase, which protect tissues by counteracting free-radical-induced oxidative damage [45]. The study’s results reveal that FVB decreases damage following cisplatin exposure by enhancing antioxidant protein levels via the Nrf2/HO-1 axis.

Membrane transport proteins are essential for the absorption and excretion of chemicals and metabolic byproducts in the liver and kidneys. OATs and OCTs are key players in renal drug secretion, with studies showing a decline in their activity as renal failure progresses. The renal proximal tubules aid in the transport and excretion of waste (drugs) through OCTs (OCT2 and OCT3) and OATs (OAT1 and OAT3) [46]. High-dose cisplatin disrupts these proteins’ transport and excretion functions, reducing their expression and leading to drug accumulation. The renal proximal tubule manages a diverse range of substances, including drugs (e.g., diuretics), uremic toxins (e.g., indoxyl sulfate), environmental toxins (e.g., mercury and aristolochic acid), metabolites (e.g., uric acid), dietary compounds, and signaling molecules. This management depends on multispecific transporters, such as those from the OAT and OCT subfamilies. These transporters play key roles in drug metabolism and nephrotoxicity and contribute to systemic signaling in conditions such as AKI and CKD [47]. Transporter expression decreases after AKI induced by ischemia or toxins, only to be upregulated during the recovery phase. The extent to which this affects the processing of drugs, metabolites, and toxins is unclear. Uremic toxins such as indoxyl sulfate and kynurenine, substrates of OATs, and other transporters, accumulate in knockout or transgenic models. This accumulation could damage renal tubular cells and may play a role in CKD development [48]. Cisplatin enters kidney cells through OCTs in the proximal tubules. It binds to DNA after accumulating, disrupting the template and halting both DNA synthesis and replication, which lead to DNA damage [49]. Although cells can repair milder DNA damage, severe damage is irreversible and leads to cell death. Most apoptotic cells appear in the proximal tubule, making DNA damage here a critical driver of cisplatin nephrotoxicity [46]. The literature highlights OCTs’ involvement in cisplatin-induced nephrotoxicity as they regulate key proteins associated with fibrosis, inflammation, and nutrient sensing. OCT1/2 knockout mice are partially protected from cisplatin-induced kidney and nerve damage [50]. OAT1 and OAT3 play crucial roles in clearing organic anions, including toxins and drugs, in proximal tubules. A reduction in cisplatin nephrotoxicity was observed in Oat1-knockout mice, despite its association with cation transporters. The mechanism suggests that extrarenal metabolism generates glutathione and cysteine conjugates, which act as substrates for OAT transporters. Given that OAT1 and OAT3 are not involved in cisplatin transport, their contributions to its nephrotoxicity remain undetermined [51]. By restoring the levels of OCT2, OCT3, OAT1, and OAT3 in kidney tubules, the FVB extract contributed to normalizing drug transport and excretion, ultimately improving drug clearance.

*F. velutipes* Singer, a mushroom rich in polysaccharides, phenols, flavonoids, and terpenes, is consumed in large amounts; however, the brown strain is recent, and its renoprotective effects and composition require further investigation [20]. Just as humans consume *F. velutipes*, water is used for extraction. Many natural products, including flavonoids, terpenes, saponins, polyphenols, and polysaccharides, have been shown to affect pathways related to cisplatin-induced CKD [49]. Our study revealed that the FVB extract contains gallic acid and quercetin. Previous research has demonstrated the kidney-protective benefits of these polyphenols. Gallic acid increases kidney weight and glomerular volume, inhibits MMP-2, elevates SOD, and reduces MDA levels [52]. Quercetin effectively reduces the oxidative stress, inflammation, and mitochondrial dysfunction associated with cisplatin-induced CKD while preventing CKD in mesangial cells by modulating the TGF-β1/SMAD pathway [53].

## 4. Materials and Methods

### 4.1. Sample Preparation

The FVB used in this study was cultivated by the Wanshen mushroom farm in Nantou, Taiwan. The bottom 2 cm of the mushroom was removed, and the upper part of the fruiting body was retained and mixed with distilled water at a mushroom-to-water weight ratio of 1:4 (*w*/*w*). The mixture was extracted at 121 °C and 1.5 kg/cm^2^ for 15 min, and this process was performed twice. The extract was filtered using a cloth press to remove residue. The filtrate was then concentrated under reduced pressure in a 50 °C water bath (PANCHUM, Kaohsiung, Taiwan) to obtain a fluid extract, which was subsequently lyophilized (Kingmech, New Taipei, Taiwan) to remove the water and yield the test sample. The sample was stored at −20 °C. The extraction yield was 2.92%. Concentrations of 0.5 g/kg and 1.0 g/kg were prepared for the animal experiments.

### 4.2. Chemicals and Reagents

Cisplatin, gallic acid, quercetin, and NAC were purchased from Sigma-Aldrich (St. Louis, MO, USA). The BUN and CRE detection kits were obtained from HUMAN Diagnostics Worldwide (Wiesbaden, Germany). The enzyme-linked immunosorbent assay (ELISA) kits for TNF-α, IL-1β, and IL-6 were procured from BioLegend (San Diego, CA, USA). The antibodies against p-p38, p38, Bax, Nrf2, HO-1, Keap1, GSK3β, p-GSK3β, and WNT2 were obtained from Abcam (Cambridge, UK). The antibodies against SOD-1 were purchased from BioVision (Milpitas Blvd, Milpitas, CA, USA). The antibodies against JNK, Bcl-2, Beclin-1, LC3-I/II, and p62 were purchased from Cell Signaling (Danvers, MA, USA). The antibodies against p-PI3K were purchased from Elabscience (Memorial Drive, Suite, Houston, TX, USA). The antibodies against iNOS, COX-2, p-AKT, AKT, p-NF-κB, NF-κB, p-ERK, p-JNK, caspase-3, TLR4, p-IκBα, IκBα, CAT, GPx3, fibronectin, collagen, α-SMA, and MMP2 were purchased from GeneTex (Alton Pkwy Irvine, CA, USA). The antibodies against PI3K and ERK were purchased from Invitrogen (Waltham, MA, USA). The antibodies against β-actin and MMP9 were purchased from Millipore (Billerica, MA, USA). The antibodies against p53 were purchased from Bioss Antibodies (Trade Center, Suite Woburn, MA, USA). The antibodies against TGF-β, p-Smad3, Smad3, OAT1, and OAT3 were obtained from ABclonal (Woburn, MA, USA). Antibodies against OCT2 were purchased from Proteintech (LubioScience GmbH, Baumackerstrasse, Zürich, Switzerland). Finally, the antibodies against OCT3 were purchased from Taiclone (Taipei, Taiwan).

### 4.3. Experimental Animal Design

Male C57BL/6 mice (6 weeks old, weighing approximately 21–23 g) were procured from BioLASCO Taiwan (Taipei, Taiwan) and kept under the following environmental conditions: a 12-hour light/dark cycle, temperature of 23 °C, and relative humidity of 50%. The animals were maintained according to the standard animal protocol approved by the China Medical University (CMUIACUC-2019-377). The mice were provided with a 1-week acclimation period and divided into five groups: (1) The N group was the control group; mice in this group received a normal diet and water with no interventions throughout the experiment. (2) In the Cis group, CKD was induced through intraperitoneal injections of 7.5 mg/kg cisplatin once in the first, second, and fourth weeks. (3) In the Cis + NAC group (positive control), the mice received intraperitoneal injections of 7.5 mg/kg cisplatin once in the first, second, and fourth weeks, and from the fourth to the sixth week, 500 mg/kg NAC was administered orally by gavage for 14 consecutive days. (4) In the Cis + GL group, 7.5 mg/kg cisplatin was administered once in the first, second, and fourth weeks, and from the fourth to the sixth week, the mice received 0.5 g/kg FVB extract orally by gavage for 14 consecutive days. (5) In the Cis + GH group, 7.5 mg/kg cisplatin was administered once in the first, second, and fourth weeks, and from the fourth to the sixth week, the mice received 1.0 g/kg FVB extract orally by gavage for 14 consecutive days. On day 42, in the sixth week, the mice were euthanized, and blood and kidney samples were collected for subsequent experimental analysis [54].

### 4.4. Body Mass and Kidney/Body Mass Ratio

All mice were weighed before each intraperitoneal injection and oral administration. Body mass was recorded twice weekly in grams. After the sixth week, following the sacrifice of the mice, kidney mass was recorded in grams. The kidney mass/body mass ratio for a mouse was calculated by dividing the kidney mass by the final recorded body mass. To calculate the kidney/body weight ratio, the equation (kidney weight/body weight) × 100% was applied [54].

### 4.5. Histopathological Analysis and Scoring

Kidney cross-sections from near the renal pelvis, measuring 0.2–0.3 cm, were soaked in 10% formalin solution. The tissue samples were sent to Professor Liao Jiunn-Wang’s laboratory at the Graduate Institute of Veterinary Pathobiology, National Chung Hsing University, where they underwent histopathological staining and analysis using H&E staining and Masson’s trichrome staining followed by histopathological scoring. Renal injury was assessed based on the percentage of epithelial necrosis in cortical tubules, with scores as follows: 0 = normal kidney; 1 = less than 25% damage; 2 = 25–50% damage; 3 = 50–75% damage; and 4 = more than 75% damage [54].

### 4.6. Renal Function Analysis: BUN and CRE

The blood samples were centrifuged (KUBOTA, Osaka, Japan) at 4 °C and 3500 rpm for 20 min. The supernatants were collected and sent to a laboratory for analysis.

### 4.7. GSH Analysis

Kidney samples were homogenized in 10% trichloroacetic acid solution and centrifuged (Kubota, Osaka, Japan) at 4 °C and 3000 rpm for 15 min. A mixture of 100 μL of 5,5′-dithiobis-(2-nitrobenzoic acid) solution and 9900 μL of 0.25 M Tris buffer was prepared, and 150 μL was added to each well of a 96-well plate. Supernatants from the homogenized samples (50 μL each) were added in duplicate. After the wells were incubated in the dark for 10 min, the absorbance was measured at OD = 412 nm using a microplate spectrophotometer (VersaMax, Molecular Devices, LLC., San Jose, CA, USA) and the absorbance data were analyzed [55].

### 4.8. MDA Analysis

Kidney samples were mixed with 10 × 50 mM sodium phosphate buffer solution (pH 7.4) and centrifuged at 4 °C and 10,000 rpm for 10 min (KUBOTA, Osaka, Japan). To 400 µL of the supernatant, 100 µL of 0.2% butylated hydroxytoluene was added, followed by 400 µL of 0.4% tert-butyl alcohol coloring agent. The mixture was vortexed for approximately 15 s and heated in a dry bath (Kaltis, New Taipei, Taiwan) at 90 °C–95 °C for 45 min. After it was subjected to a 5 min ice bath, 900 µL of n-butanol (1:11 ratio) was added and mixed. The samples were centrifuged again at 4 °C and 10,000 rpm for 10 min. Finally, 200 µL of the supernatant was placed in triplicate in the wells of a 96-well plate, and absorbance was measured at OD = 535 nm using a microplate spectrophotometer (VersaMax, Molecular Devices, LLC., San Jose, CA, USA) [56].

### 4.9. Nitrite Analysis

A total of 100 µL of serum was placed into each well of a 96-well plate, and 50 µL of Griess Reagent was then added. The reagent was prepared by mixing N-(1-naphthyl) ethylenediamine in H_2_O with 1% sulfanilamide in 5% phosphoric acid at a 1:1 ratio. The absorbance was measured at OD = 540 nm using a microplate spectrophotometer [57].

### 4.10. Inflammatory Factor Analysis

The pro-inflammatory cytokines IL-1β, IL-6, and TNF-α were quantified using ELISA kits, and the absorbance was measured at 450 nm using a microplate reader.

### 4.11. Western Blotting

Kidney samples were mixed with 10× phosphate-buffered saline homogenization buffer and centrifuged at 4 °C and 10,000 rpm for 15 min. The supernatants were collected and their concentrations of proteins were measured. The samples were heated in a dry bath at 105 °C for 5 min and then cooled on ice for 5 min. To conduct electrophoresis, wells of electrophoresis gel were loaded with 10 μL of sample in triplicate. Electrophoresis was performed using pH 8.3 running buffer (Bio-Rad, Hercules, CA, USA), with the initial conditions of 100 V for 10 min followed by 130 V for 70–90 min. The resultant proteins were transferred to a membrane using a transfer buffer (BioRAD, USA) at 130 V for 65–75 min. The membrane (Millipore, Billerica, MA, USA) was blocked in milk–TBST (1:10) solution and incubated with the primary antibody overnight. After being washed, the membrane was incubated with the secondary antibody at room temperature for 1 h. An enhanced chemiluminescence detection reagent was added and mixed in for 2 min. Images were captured using a Kodak Molecular imaging system (USA) and analyzed with AlphaEase FC software (Version 4.0.5, Eastman Kodak Company, Rochester, NY, USA).

### 4.12. HPLC

HPLC was performed using the following mobile phases: A, acetonitrile; B, 0.5% acetic acid; and C, methanol. Samples of the FVB extract were prepared at concentrations of 10,000 ppm. Standard solutions of quercetin and gallic acid were prepared at concentrations of 100 ppm. The samples and standards were filtered through sterile syringe filters (0.22 μm), and 1.5–2.0 mL was collected in vials. The HPLC system (Hitachi-High Technologies, Tokyo, Japan) was set with the following conditions: temperature of 35 °C, wavelength of 280 nm, and flow rate of 1.0 mL/min. The mobile phase gradient elution schedule was as follows: 0–20 min, 2–10% (A) and 98–90% (B); 20–30 min, 10–25% (A) and 90–75% (B); 30–40 min, 25–35% (A) and 75–65% (B); and 40–50 min, 35–2% (A) and 65–98% (B). Data were collected and analyzed.

### 4.13. Statistical Analysis

The statistical analyses were conducted using SPSS version 22. The results are displayed as the means ± SD for each group. The data were subjected to one-way analysis of variance (ANOVA), followed by Scheffé’s post hoc test for further comparison. Differences were considered significant when the *p*-value was lower than 0.05 (indicated by *), highly significant when less than 0.01 (indicated by **), and extremely significant when less than 0.001 (indicated by ***).

## 5. Conclusions

**Figure 10:**
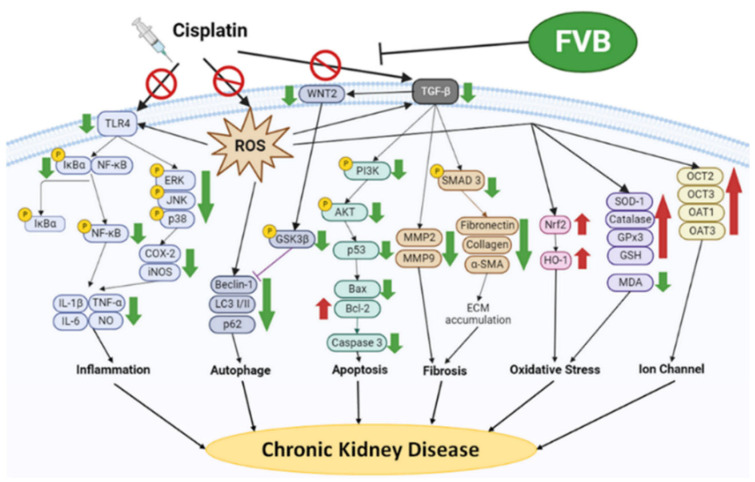
A scheme displaying the protective effect of FVB against cisplatin-induced CDK. The green arrows denote increases and the red arrows denote decreases. Forbidden symbol denote suppression.

In this study, the FVB extract was discovered to have significant ameliorative effects on cisplatin-induced nephrotoxicity in CKD experiments. The qualitative HPLC analysis confirmed that the extract contained the following two bioactive components: gallic acid and quercetin. The tests on blood and kidney samples revealed that a high dose of the FVB extract (dose of 1 kg/kg) more significantly improved cisplatin-induced nephrotoxicity than the clinically used adjuvant NAC in terms of renal function, kidney tissue structure, and various pathways of kidney injury. The FVB extract inhibited TLR4; reduced IκBα- and NF-κB-induced secretion of the proinflammatory factors IL-1β, IL-6, TNF-α, and NO and decreased phosphorylation of the MAPK family members ERK, JNK, and p38. These actions indirectly reduced the production of COX-2 and iNOS, blocking the exacerbation of inflammatory pathways, mitigating oxidative stress, and assisting in the reduction in TGF-β/SMAD3-related profibrotic substances in kidney cells. The FVB extract promoted expression of the antioxidant proteins Nrf2 and HO-1; increased the levels of catalase, SOD-1, GSH, and GPx3; and facilitated the removal of excessive cisplatin in the kidneys. Compared with NAC, the FVB extract more effectively inhibited inflammatory factors, fibrotic pathways, and ion transport proteins (Figure 10). The results of this study provide a basis for further exploration of the physiological activities and other potential effects of FVB extract. FVB extract may become a clinically important auxiliary food for improving the nephrotoxic side effects of drug therapy.

## Figures and Tables

**Table 1:** Initial and final body masses, kidney masses, and kidney indexes of mice with cisplatin-induced CKD. Kidney index was calculated as the kidney mass in milligrams divided by the final body mass in grams. Values are presented as the means ± SD (*n* = 6). ^###^ *p* < 0.001, significant difference between the Cis and N groups; * *p* < 0.05, ** *p* < 0.01, and *** *p* < 0.001, significant differences between the Cis group and Cis + NAC, Cis + GL, and Cis + GH groups.

Group (*n* = 6)	Dosage (g/kg)	Body Weight (g)		Renal Weight (g)	Kidney Index (mg/g)
		Initial	Final		
N	-	22.2 ± 0.76	25.9 ± 1.04	0.30 ± 0.02	1.18 ± 0.06
Cis	0.0075	21.8 ± 0.58	14.3 ± 1.70 ^###^	0.14 ± 0.01 ^###^	0.86 ± 0.06 ^###^
Cis + NAC	0.5	22.5 ± 0.55	16.4 ± 1.25 *	0.17 ± 0.01 **	1.07 ± 0.02 ***
Cis + GH	1.0	23.4 ± 0.87	20.3 ± 2.50 ***	0.20 ± 0.01 ***	1.19 ± 0.15 ***
Cis + GL	0.5	22.3 ± 0.75	17.6 ± 1.76 **	0.16 ± 0.02 *	1.05 ± 0.11 **

## Data Availability

Data are contained within the article.

## Supplementary Materials

The following supporting information can be downloaded at: https://www.mdpi.com/article/10.3390/ijms252212096/s1.

## Abbreviations

KD: chronic kidney disease; FVB: brown-strain *Flammulina velutipes;* NAC: N-acetylcysteine; HPLC; high-performance liquid chromatography; CRE: creatinine; BUN: blood urea nitrogen; ROS: reactive oxygen species; MAPK: mitogen-activated protein kinase; JNK: C-jun NH2-terminal kinase; ERK: extracellular-signal-regulated kinase; NF-κB: nuclear factor of κB; HO-1: heme oxygenase 1; GPx3: glutathione peroxidases 3; Nrf2: nuclear factor erythroid 2–related factor 2; SOD-1: superoxide dismutase 1; IκB-α: inhibitor of the nuclear factor kappa B; TLR-4: toll-like receptor 4; COX-2: cyclooxygenase-2; iNOS: inducible nitric oxide synthase; Bcl-2: B-cell lymphoma 2; Bax: Bcl-2-associated X; TNF-α: tumor necrosis factor-α; IL-1ß: interleukin-1β; IL-6: interleukin-6; TGF-β: transforming growth factor beta; SMAD3: mothers against decapentaplegic homolog 3; MMP2: matrix metalloproteinase-2; MMP9: matrix metalloproteinase-9; OCT2: organic cation transporter 2; OCT3: organic cation transporter 3; OAT1: organic anion transporter 1; OAT3: organic anion transporter 3; WNT2: Wnt family member 2.

## References

[1] Gur C., Kandemir F.M., Caglayan C., Satıcı E. (2022). Chemopreventive effects of hesperidin against paclitaxel-induced hepatotoxicity and nephrotoxicity via amendment of Nrf2/HO-1 and caspase-3/Bax/Bcl-2 signaling pathways. Chem. Biol. Interact..

[2] Loren P., Lugones Y., Saavedra N., Saavedra K., Páez I., Rodriguez N., Moriel P., Salazar L.A. (2022). MicroRNAs involved in intrinsic apoptotic pathway during cisplatin-induced nephrotoxicity: Potential use of natural products against DDP-induced apoptosis. Biomolecule.

[3] Aladaileh S.H., Al-Swailmi F.K., Abukhalil M.H., Ahmeda A.F., Mahmoud A.M. (2021). Punicalagin prevents cisplatin-induced nephrotoxicity by attenuating oxidative stress, inflammatory response, and apoptosis in rats. Life Sci..

[4] Khine H.E.E., Ecoy G.A.U., Roytrakul S., Phaonakrop N., Pornputtapong N., Prompetchara E., Chanvorachote P., Chaotham C. (2021). Chemosensitizing activity of peptide from *Lentinus squarrosulus* (Mont.) on cisplatin-induced apoptosis in human lung cancer cells. Sci. Rep..

[5] Mapuskar K.A., Steinbach E.J., Zaher A., Riley D.P., Beardsley R.A., Keene J.L., Holmlund J.T., Anderson C.M., Zepeda-Orozco D., Buatti J.M. (2021). Mitochondrial superoxide dismutase in cisplatin-induced kidney injury. Antioxidants.

[6] Wang Z., Sun W., Sun X., Wang Y., Zhou M. (2020). Kaempferol ameliorates cisplatin induced nephrotoxicity by modulating oxidative stress, inflammation and apoptosis via ERK and NF-κB pathways. AMB Express.

[7] Liu H.T., Wang T.E., Hsu Y.T., Chou C.C., Huang K.H., Hsu C.C., Liang H.J., Chang H.W., Lee T.H., Tsai P.S. (2019). Nanoparticulated honokiol mitigates cisplatin-induced chronic kidney injury by maintaining mitochondria antioxidant capacity and reducing caspase 3-associated cellular apoptosis. Antioxidants.

[8] Szefler B., Czeleń P., Kruszewski S., Siomek-Górecka A., Krawczyk P. (2022). The assessment of physicochemical properties of Cisplatin complexes with purines and vitamins B group. J. Mol. Graph. Model..

[9] Ismail R.S., El-Awady M.S., Hassan M.H. (2020). Pantoprazole abrogated cisplatin-induced nephrotoxicity in mice via suppression of inflammation, apoptosis, and oxidative stress. Naunyn Schmiedeberg’s Arch. Pharmacol..

[10] Miller R.P., Tadagavadi R.K., Ramesh G., Reeves W.B. (2010). Mechanisms of cisplatin nephrotoxicity. Toxins.

[11] Wei Q., Dong G., Yang T., Megyesi J., Price P.M., Dong Z. (2007). Activation and involvement of p53 in cisplatin-induced nephrotoxicity. Am. J. Physiol. Ren. Physiol..

[12] Imig J.D., Ryan M.J. (2013). Immune and inflammatory role in renal disease. Compr. Physiol..

[13] Landau S.I., Guo X., Velazquez H., Torres R., Olson E., Garcia-Milian R., Moeckel G.W., Desir G.V., Safirstein R. (2019). Regulated necrosis and failed repair in cisplatin-induced chronic kidney disease. Kidney Int..

[14] Ma N., Wei Z., Hu J., Gu W., Ci X. (2021). Farrerol ameliorated cisplatin-induced chronic kidney disease through mitophagy induction via Nrf2/PINK1 Pathway. Front. Pharmacol..

[15] Song A., Zhang C., Meng X. (2021). Mechanism and application of metformin in kidney diseases: An update. Biomed. Pharmacother..

[16] Tsai Y.S., Chen Y.P., Lin S.W., Chen Y.L., Chen C.C., Huang G.J. (2022). *Lactobacillus rhamnosus* GKLC1 ameliorates cisplatin-induced chronic nephrotoxicity by inhibiting cell inflammation and apoptosis. Biomed. Pharmacother..

[17] Hu Y.N., Sung T.J., Chou C.H., Liu K.L., Hsieh L.P., Hsieh C.W. (2019). Characterization and antioxidant activities of yellow strain *Flammulina velutipes* (Jinhua Mushroom) polysaccharides and their effects on ROS content in L929 Cell. Antioxidants.

[18] Shen P., Lin W., Deng X., Ba X., Han L., Chen Z., Qin K., Huang Y., Tu S. (2021). Potential implications of quercetin in autoimmune diseases. Front. Immunol..

[19] Wianowska D., Olszowy-Tomczyk M. (2023). A concise profile of gallic acid from its natural sources through biological properties and chemical methods of determination. Molecule.

[20] Chou Y.N., Lee M.M., Deng J.S., Jiang W.P., Lin J.G., Huang G.J. (2023). Water extract from brown strain of *Flammulina velutipes* alleviates cisplatin-induced acute kidney injury by attenuating oxidative stress, inflammation, and autophagy via PI3K/AKT pathway regulation. Int. J. Mol. Sci..

[21] Zhang Y., Qin S., Song Y., Yuan J., Hu S., Chen M., Li L. (2022). Alginate oligosaccharide alleviated cisplatin-induced kidney oxidative stress via Lactobacillus genus-FAHFAs-Nrf2 Axis in mice. Front. Immunol..

[22] Deng J.S., Jiang W.P., Chen C.C., Lee L.Y., Li P.Y., Huang W.C., Liao J.C., Chen H.Y., Huang S.S., Huang G.J. (2020). *Cordyceps cicadae* mycelia ameliorate cisplatin-induced acute kidney injury by suppressing the TLR4/NF-κB/MAPK and activating the HO-1/Nrf2 and Sirt-1/AMPK pathways in mice. Oxid. Med. Cell. Longev..

[23] Yu X., Meng X., Xu M., Zhang X., Zhang Y., Ding G., Huang S., Zhang A., Jia Z. (2018). Celastrol ameliorates cisplatin nephrotoxicity by inhibiting NF-κB and improving mitochondrial function. EBioMedicin.

[24] Kwon D.J., Ju S.M., Youn G.S., Choi S.Y., Park J. (2013). Suppression of iNOS and COX-2 expression by flavokawain A via blockade of NF-κB and AP-1 activation in RAW 264.7 macrophages. Food Chem. Toxicol..

[25] Yin Q., Xiong H. (2022). Chemotherapy-induced nephrotoxicity was improved by crocin in mouse model. Eur. J. Histochem..

[26] Fu S., Hu X., Ma Z., Wei Q., Xiang X., Li S., Wen L., Liang Y., Dong Z. (2022). P53 in proximal tubules mediates chronic kidney problems after cisplatin treatment. Cell.

[27] Liang S., Wu Y.S., Li D.Y., Tang J.X., Liu H.F. (2022). Autophagy and Renal Fibrosis. Aging Dis..

[28] Li L., Zepeda-Orozco D., Black R., Lin F. (2010). Autophagy is a component of epithelial cell fate in obstructive uropathy. Am. J. Pathol..

[29] Teh Y.M., Mualif S.A., Lim S.K. (2022). A comprehensive insight into autophagy and its potential signaling pathways as a therapeutic target in podocyte injury. Int. J. Biochem. Cell Biol..

[30] Cai Y., Yang W., Pan M., Wang C., Wu W., Zhu S. (2018). Wnt2 knock down by RNAi inhibits the proliferation of in vitro-cultured human keloid fibroblasts. Medicine.

[31] Wu D., Pan W. (2010). GSK3: A multifaceted kinase in Wnt signaling. Trends Biochem. Sc..

[32] Pal S.K., Quinn D.I. (2013). Differentiating mTOR inhibitors in renal cell carcinoma. Cancer Treat. Rev..

[33] Yin H., Zuo Z., Yang Z., Guo H., Fang J., Cui H., Ouyang P., Chen X., Chen J., Geng Y. (2021). Nickel induces autophagy via PI3K/AKT/mTOR and AMPK pathways in mouse kidney. Ecotoxicol. Enviro. Saf..

[34] Wang J., Wang Y., Liu Y., Cai X., Huang X., Fu W., Wang L., Qiu L., Li J., Sun L. (2022). Ferroptosis, a new target for treatment of renal injury and fibrosis in a 5/6 nephrectomy-induced CKD rat model. Cell Death Discov..

[35] Jia M., Qiu H., Lin L., Zhang S., Li D., Jin D. (2022). Inhibition of PI3K/AKT/mTOR signaling pathway activates autophagy and suppresses peritoneal fibrosis in the process of peritoneal dialysis. Front. Physiol..

[36] Li B., Ji Y., Yi C., Wang X., Liu C., Wang C., Lu X., Xu X., Wang X. (2022). Rutin inhibits Ox-LDL-mediated macrophage inflammation and foam cell formation by inducing autophagy and modulating PI3K/ATK signaling. Molecule.

[37] Li Y.Y., Tian Z.H., Pan G.H., Zhao P., Pan D.J., Zhang J.Q., Ye L.Y., Zhang F.R., Xu X.D. (2022). Heidihuangwan alleviates renal fibrosis in rats with 5/6 nephrectomy by inhibiting autophagy. Front. Pharm..

[38] Peng J., Xiao X., Li S., Lyu X., Gong H., Tan S., Dong L., Sanders Y.Y., Zhang X. (2023). Aspirin alleviates pulmonary fibrosis through PI3K/AKT/mTOR-mediated autophagy pathway. Exp. Gerontol..

[39] Wozniak J., Floege J., Ostendorf T., Ludwig A. (2021). Key metalloproteinase-mediated pathways in the kidney. Nat. Rev. Nephrol..

[40] Andreucci M., Provenzano M., Faga T., Michael A., Patella G., Mastroroberto P., Serraino G.F., Bracale U.M., Ielapi N., Serra R. (2021). Aortic aneurysms, chronic kidney disease and metalloproteinases. Biomolecule.

[41] Uddin M.J., Kim E.H., Hannan M.A., Ha H. (2021). Pharmacotherapy against oxidative stress in chronic kidney disease: Promising small molecule natural products targeting Nrf2-HO-1 signaling. Antioxidant.

[42] Mahran Y.F., Hassan H.M. (2020). *Ganoderma lucidum* prevents cisplatin-induced nephrotoxicity through inhibition of epidermal growth Factor receptor signaling and autophagy-mediated apoptosis. Oxid. Med. Cell Longev..

[43] Guerrero-Hue M., Rayego-Mateos S., Vázquez-Carballo C., Palomino-Antolín A., García-Caballero C., Opazo-Rios L., Morgado-Pascual J.L., Herencia C., Mas S., Ortiz A. (2020). Protective Role of Nrf2 in Renal Disease. Antioxidants.

[44] Joo C.R., Cheng M.S., Shik K.Y. (2014). Desoxyrhapontigenin up-regulates Nrf2-mediated heme oxygenase-1 expression in macrophages and inflammatory lung injury. Redox Biol..

[45] Sahin K., Tuzcu M., Gencoglu H., Dogukan A., Timurkan M., Sahin N., Aslan A., Kucuk O. (2010). Epigallocatechin-3-gallate activates Nrf2/HO-1 signaling pathway in cisplatin-induced nephrotoxicity in rats. Life Sci..

[46] Nigam S.K., Wu W., Bush K.T., Hoenig M.P., Blantz R.C., Bhatnagar V. (2015). Handling of drugs, metabolites, and uremic toxins by kidney proximal tubule drug transporters. Clin. J. Am. Soc. Nephrol..

[47] Shi B., Zhang Y., Huang B., Lin H., Zhou Q., Wang Y., Cai Z., Liu M. (2022). The system profile of renal drug transporters in tubulointerstitial fibrosis model and consequent effect on pharmacokinetics. Molecule.

[48] Susa K., Kobayashi K., Galichon P., Matsumoto T., Tamura A., Hiratsuka K., Gupta N.R., Yazdi I.K., Bonventre J.V., Morizane R. (2023). ATP/ADP biosensor organoids for drug nephrotoxicity assessment. Front. Cell Dev. Bio.

[49] Ma S., Zhang H., Xu J. (2021). Characterization, antioxidant and anti-Inflammation capacities of fermented *Flammulina Velutipes* Polyphenols. Molecules.

[50] Franke R.M., Kosloske A.M., Lancaster C.S., Filipski K.K., Hu C., Zolk O., Mathijssen R.H., Sparreboom A. (2010). Influence of Oct1/Oct2-deficiency on cisplatin-induced changes in urinary N-acetyl-beta-D-glucosaminidase. Clin. Cancer Res..

[51] Nieskens T.T.G., Peters J.G.P., Dabaghie D., Korte D., Jansen K., Van Asbeck A.H., Tavraz N.N., Friedrich T., Russel F.G.M., Masereeuw R. (2018). Expression of organic anion transporter 1 or 3 in human kidney proximal tubule cells reduces cisplatin sensitivity. Drug Metab. Dispos..

[52] Peng C.C., Hsieh C.L., Wang H.E., Chung J.Y., Chen K.C., Peng R.Y. (2012). Ferulic acid is nephrodamaging while gallic acid is renal protective in long term treatment of chronic kidney disease. Clin. Nutr..

[53] Widowati W., Prahastuti S., Tjokropranoto R., Onggowidjaja P., Kusuma H.S.W., Afifah E., Arumwardana S., Maulana M.A., Rizal R. (2022). Quercetin prevents chronic kidney disease on mesangial cells model by regulating inflammation, oxidative stress, and TGF-beta1/SMADs pathway. PeerJ.

[54] Chien L.H., Wu C.T., Deng J.S., Jiang W.P., Huang W.C., Huang G.J. (2021). Salvianolic acid C protects against cisplatin-induced acute kidney injury through attenuation of inflammation, oxidative stress and apoptotic effects and activation of the CaMKK–AMPK–Sirt1-associated signaling Pathway in mouse models. Antioxidant.

[55] Chien L.H., Deng J.S., Jiang W.P., Chou Y.N., Lin J.G., Huang G.J. (2023). Evaluation of lung protection of *Sanghuangporus sanghuang* through TLR4/NF-κB/MAPK, keap1/Nrf2/HO-1, CaMKK/AMPK/Sirt1, and TGF-β/SMAD3signaling pathways mediating apoptosis and autophagy. Biomed. Pharmacother..

[56] Lin W.H., Jiang W.P., Chen C.C., Lee L.Y., Tsai Y.S., Chien L.H., Chou Y.N., Deng J.S., Huang G.J. (2022). Renoprotective effect of *Pediococcus acidilactici* GKA4 on cisplatin-induced acute kidney injury by mitigating inflammation and oxidative stress and regulating the MAPK, AMPK/SIRT1/NF-κB, and PI3K/AKT pathways. Nutrients.

[57] Jiang W.P., Deng J.S., Huang S.S., Wu S.H., Chen C.C., Liao J.C., Chen H.Y., Lin H.Y., Huang G.J. (2021). *Sanghuangporus sanghuang* mycelium prevents paracetamol-induced hepatotoxicity through regulating the MAPK/NF-κB, Keap1/Nrf2/HO-1, TLR4/PI3K/Akt, and CaMKKβ/LKB1/AMPK pathways and suppressing oxidative stress and inflammation. Antioxidants.

