# Supplementary material for: Renoprotective Effects of Brown-Strain Flammulina velutipes Singer in Chronic Kidney Disease-Induced Mice Through Modulation of Oxidative Stress and Inflammation and Regulation of Renal Transporters
Source: Int J Mol Sci. 2024 Nov 11;25(22):12096. doi: 10.3390/ijms252212096 (PMC11593982; doi:10.3390/ijms252212096)
Supplement: Supplementary file 1 [file ijms-25-12096-s001.zip › ijms-3276415-supplementary.pdf]

Effect of FVB on Body Mass in Mice with Cisplatin-Induced Nephrotoxicity

The animals in the experiment were allocated to one of five groups: the normal (N), cisplatin (Cis), cisplatin + N-acetylcysteine (NAC; the positive control) (Cis + NAC), cisplatin + low-dose FVB extract (Cis + GL), and cisplatin + high-dose FVB extract (Cis + GH) groups. In the 6th week of the experiment, the body mass of the cisplatin group (Cis group) was significantly lower than that of the normal group, with the average difference being 11.6 g ($p < 0.001$). The body mass of the Cis + NAC group was 2.03 g higher on average ($p < 0.05$) and that of the Cis + GL group was 3.28 g higher on average ($p < 0.01$) than that of the Cis group. The average body mass of the Cis + GH group was 6.01 g higher compared to the Cis group ($p <$

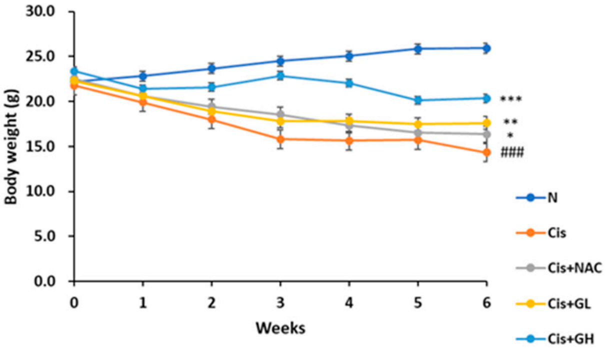

0.001). In addition, the body weight of the Cis + GH group was 3.98g higher than that of the Cis + NAC group ($p < 0.01$; Figure 1).

Figure S1. Weekly average body mass of mice with cisplatin (Cis)-induced CKD. Values are presented as mean ± standard deviation (SD; n = 6). ###$p < 0.001$, significant difference between Cis and N groups; *$p < 0.05$, **$p < 0.01$, and ***$p < 0.001$, significant differences between Cis group and Cis + N-acetylcysteine (NAC), Cis + low-dose FVB extract (GL), and Cis + high-dose FVB extract (GH) groups.
